# Combination of curcumin and bicalutamide enhanced the growth inhibition of androgen-independent prostate cancer cells through SAPK/JNK and MEK/ERK1/2-mediated targeting NF-κB/p65 and MUC1-C

**DOI:** 10.1186/s13046-015-0168-z

**Published:** 2015-05-15

**Authors:** Jing Li, SongTao Xiang, QiouHong Zhang, JingJing Wu, Qing Tang, JianFu Zhou, LiJun Yang, ZhiQiang Chen, Swei Sunny Hann

**Affiliations:** Laboratory of Tumor Biology, Guangdong Provincial Hospital of Chinese Medicine, The Second Clinical Medical Collage, University of Guangzhou Traditional Chinese Medicine, Guangzhou, Guangdong Province 510120 China; Department of Urology Surgery, Guangdong Provincial Hospital of Chinese Medicine, The Second Clinical Medical Collage, University of Guangzhou Traditional Chinese Medicine, Guangzhou, Guangdong Province 510120 China; Higher Education Mega Center, Panyu District, Guangdong Provincial Hospital of Chinese Medicine, No. 55, Neihuan West Road, Guangzhou, Guangdong Province 510006 People’s Republic of China

**Keywords:** Androgen-independent prostate cancer cells, Curcumin, Bicalutamide, SAPK/JNK, NF-κB/p65, MUC1-C

## Abstract

**Background:**

Prostate cancer is one of the most common malignancies in men. The mucin 1 (MUC1) heterodimeric oncoprotein is overexpressed in human prostate cancers with aggressive pathologic and clinical features, resulting in a poor outcome. However, the functional role for MUC1 C-terminal domain (MUC1-C) in androgen-independent prostate cancer occurrence and development has remained unclear.

**Methods:**

Cell viability was measured by MTT assays. Western blot analysis was performed to measure the phosphorylation and protein expression of SAPK/JNK and ERK1/2, and MUC1-C, NF-κB subunit p65 and p50. Exogenous expression of MUC1-C, NF-κB subunit p65 was carried out by transient and electroporated transfection assays.

**Results:**

We showed that curcumin inhibited the growth of androgen-independent prostate cancer cells and a synergy was observed in the presence of curcumin and bicalutamide, the androgen receptor antagonist. To further explore the potential mechanism underlining this, we found that curcumin increased the phosphorylation of ERK1/2 and SAPK/JNK, which was enhanced by bicalutamide. In addition, curcumin reduced the protein expression of MUC1-C and NF-κB subunit p65, which were abrogated in the presence of the inhibitors of MEK/ERK1/2 (PD98059) and SAPK/JNK (SP60015). A further reduction was observed in the combination of curcumin with bicalutamide. Moreover, while exogenous expression of MUC1-C had little effect on curcumin-reduced p65, the overexpression of p65 reversed the effect of curcumin on MUC1-C protein expression suggesting that p65 is upstream of MUC1-C. Intriguingly, we showed that exogenous expression of MUC1-C feedback diminished the effect of curcumin on phosphorylation of ERK1/2 and SAPK/JNK, and antagonized the effect of curcumin on cell growth.

**Conclusion:**

Our results show that curcumin inhibits the growth of androgen-independent prostate cancer cells through ERK1/2- and SAPK/JNK-mediated inhibition of p65, followed by reducing expression of MUC1-C protein. More importantly, there are synergistic effects of curcumin and bicalutamide. The negative feedback regulatory loop of MUC1-C to ERK1/2 and SAPK/JNK further demonstrates the role of MUC1-C that contributes to the overall responses of curcumin. This study unveils the potential molecular mechanism by which combination of curcumin with bicalutamide enhances the growth inhibition of androgen-independent prostate cancer cells.

## Introduction

Prostate cancer is one of the most common cancer types and the second leading cause of cancer related death in men in the world [1, 2]. The androgen deprivation therapy (ADT), which suppresses or reduces androgens binding to the androgen receptor (AR), is a well-known treatment strategy for advanced, recurrent and even metastatic prostate cancer; however, the long term therapeutic outcomes of ADT on prostate cancer remain uncertain, and are associated with considerable known adverse effects that affect the quality of life in prostate cancer patients [3]. Also, development of resistance to ADT is a major obstacle for the management of advanced prostate cancer [4]. Therapies with AR antagonists such as bicalutamide (Casodex) and androgen withdrawal initially regress tumors but development of various compensatory mechanisms including AR bypass signaling leading to tumor growth, and eventually develop more aggressive castration resistant prostate cancer (CRPC) [5]. Therefore, search for the novel therapeutic approaches based on various combinations of anticancer drugs and procedures to minimize the resistance in order to enhance the therapeutic efficacy are strongly required. Curcumin, an active natural polyphenol derived from the root of Curcuma longa, has been shown great potential as a novel therapeutic agent due to its pharmacological safety and efficacy in treating a wide variety of cancers [6, 7]. These facts, tested and confirmed in many different cancer types [8], have paved the way for research aimed at elucidating the potential beneficial effects of combining curcumin and various anti-cancer drugs in order to establish more efficient and less toxic cancer treatment modalities.

Cell surface-associated mucin 1 (MUC1) (previously known as PUM, MCKD1), a highly glycosylated transmembrane heterodimeric protein and a transmembrane member of the mucin family, is highly expressed in various human malignant tumors including prostate cancers and is correlated with a poor prognosis [9, 10]. MUC1 cytoplasmic tail can interact with many signaling pathways, and act as a co-transcription factor to activate various genes involved in tumor progression and metastasis. In many tumor types, expression of MUC1 correlates with aggressive, metastatic phenotype, limited response to therapy and poor survival [10, 11]. The MUC-1 C-terminal subunit (MUC1-C) is a single-pass transmembrane protein that interacts with receptor tyrosine kinases, such as epidermal growth factor receptor (EGFR) and others, at the cell membrane and contributes to activation of other kinase signaling pathways that induce proliferation and tumor growth [12–14]. MUC1-C also binds directly to nuclear factor NF-kappaB (NF-κB) p65 and promotes NF-κB-mediated gene transcription [9]. Thus, mucins including MUC1-C are considered important markers for early diagnosis and targeted therapy due to their unique expression pattern during cancer progression. Studies have provided substantial evidence for the involvement of transmembrane MUC1-C in altered cell signaling, tumor growth, and metastasis [15, 16].

In this study, we explored the potential mechanism by which combination of curcumin with bicalutamide in the inhibition growth of androgen-independent prostate cancer cells.

## Materials and methods

### Cell culture and chemicals

The prostate cancer cell lines (PC3, DU145, LNCaP) were obtained from the Sun Yat-sen Memorial Hospital, Sun Yat-sen University, Guangzhou, China. All cell lines have been tested and authenticated for absence of Mycoplasma, genotypes, drug response, and morphology in the Laboratory. Cells were grown in F-12K or DMEM (1:1) medium (obtained from GIBCO, Life Technologies, Grand Island, NY, USA) with supplemented 10 % fetal bovine serum. The polyclonal antibody against both MUC1 and MUC1-C (Cat No. ab109185) was obtained from Abcam (Cambridge, MA, USA). The antibodies against p65 (Cat No. 8242), total extracellular signal-regulated kinase1/2 (ERK1/2) (Cat No. 4695), stress-activated protein kinase/c-Jun N-terminal kinase (SAPK/JNK) (Cat No. 9258) and the phosphor-forms (Cat No. 4370 and 4668) were purchased from Cell Signaling Technology Inc (Beverly, MA, USA). Curcumin, PD98059 [(MAPK extracellular signaling-regulated kinase (ERK) kinase (MEK)/ERK inhibitor] and SP600125 (SAPK/JNK inhibitor) were obtained from Sigma-Aldrich (St. Louis, MO, USA).

### Western blot analysis

The detailed procedure was reported previously [17, 18]. Briefly, protein concentrations were determined by the Bio-Rad protein assay. Equal amounts of protein from whole cell lysates were solubilized in 5 × SDS-sample buffers and separated on 12 % SDS polyacrylamide gels. Membranes were incubated with antibodies against MUC1-C, p65, the phosphor and total ERK1/2, and SAPK/JNK. The membranes were washed and incubated with a secondary antibody raised against rabbit IgG conjugated to horseradish peroxidase (Cell Signaling Technology, Inc., Beverly, MA, USA). The membranes were washed again and transferred to freshly made ECL solution (Immobilon Western; Millpore, Billerica, MA, USA), followed by observing the signals under the Molecular Imager ChemiDoc XRS Gel Imagine System (Bio-Rad, Hercules, CA, USA) and documenting the results.

### Cell viability assay

Cell viability was measured using the 3-(4, 5-dimethylthiazol-2-yl)-2, 5-diphenyltetrazolium bromide (MTT) assay [18]. Briefly, prostate cancer cells were harvested, counted and seeded in a 96-well microtitre plate. The cells were treated with increasing concentrations of curcumin for up to 72 hrs. After incubation, 20 μl MTT solution (5 g/L) was added to each well and prostate cancer cells were incubated at 37 °C for additional 4 h. Supernatant was removed, then 200 μL solvent dimethyl sulfoxide was added to each well and oscillated for 10 min. Absorbance at 490 nm was determined through the use of ELISA reader (Perkin Elmer, Victor × 5, Waltham, MA, USA). Cell viability (% of control) was calculated as (absorbance of test sample/absorbance of control) × 100 %.

### Transient transfection assay

The cells were seeded in 6-well dishes and grown to 50–60 % confluence. The control or MUC1-C and p65 overexpression vectors (pCMV6-AC-MUC1, pCMV6-AC-p65) were obtained from OriGene Technologies, Inc. (Rockville, MD, USA). Briefly, cells were seeded in 6-well dishes and grown to 50–60 % confluence. For each well, 2 μg of control (pCMV6-AC) and MUC1-C and p65 plasmid DNA constructs were transfected into the cells using Lipofectamine 3000 reagent (Life Technologies, Grand Island, NY, USA) for up to 30 h based on the instruction from the provider, followed by treating with curcumin for an additional 24 or 48 h for other experiments.

### Statistical analysis

Data are presented as mean ± SD from three independent experiments with triplicates. Statistical significance was determined with Student's t test (two-tailed) comparison between two groups of data set. All statistical analyses were performed using GraphPad Prism 5 software (GraphPad Software, Inc., La Jolla, CA, USA). Asterisks shown in the figures indicate significant differences of experimental groups in comparison with the corresponding control condition (P < 0.05, see figure legends).

## Results

### The effect of curcumin and bicalutamide on growth of prostate cancer cells

**Fig. 1:**
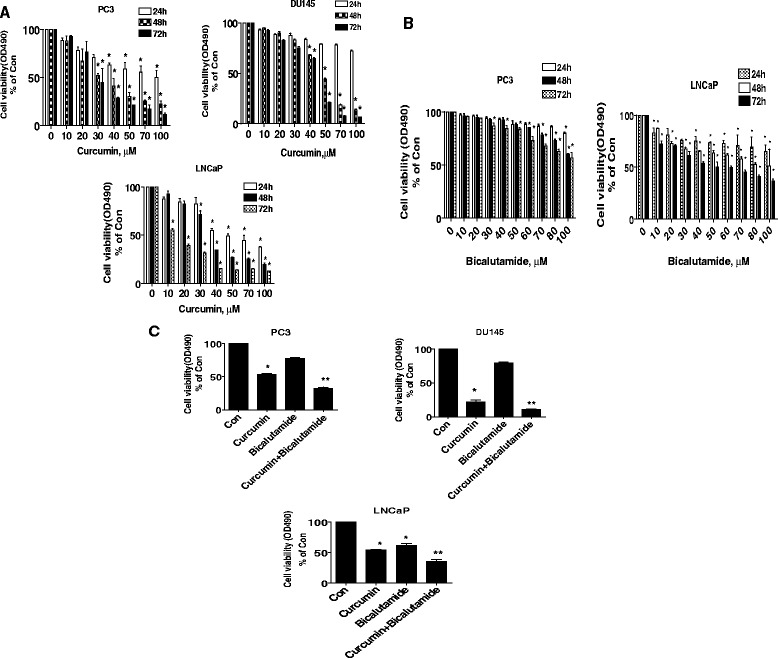
The effect of curcumin and bicalutamide on growth of prostate cancer cells. **a** Prostate cancer cells (PC3, DU145 and LNCaP) were treated with increased concentrations of curcumin for up to 72 h to examine the cell viability. **b** Prostate cancer cells (PC3 and LNCaP) were treated with increased concentrations of bicalutamide for up to 72 h to examine the cell viability **c** PC3, DU145 and LNCaP cells were treated with curcumin (40 μM) and bicalutamide (30 μM) for 48 h to examine the cell viability. The cell viability was determined using the MTT assay as described in the Materials and Methods section and was expressed as percentage of control in the mean ± SD of three separate experiments. *indicates significant difference as compared to the untreated control group (P < 0.05). **Indicates significant difference from curcumin treated alone (P < 0.05)

We first examined the effect of curcumin on prostate cancer cell growth. We showed that curcumin inhibited the growth of multiple prostate cancer cell lines in the time- and dose-dependent manner with significant effect most observed at 40 μM at 48 h (Fig. 1a). Note that low dose showing significant response were also seen in one androgen-dependent prostate cancer cell (LNCaP) compared to other two androgen-independent prostate cancer cell lines (PC3 and DU145). We also demonstrated that bicalutamide, an androgen receptor antagonist, inhibited cell growth in dose- and time-dependent fashion in PC3 and LNCaP cells. Note that the inhibitory effect of bicalutamid was different in LNCaP and PC3 cells; lower dose and shorter time were observed in LNCaP cells (10 μM at 24 h), whereas high doses and longer time were found in PC3 (30 μM, 72 h) (Fig. 1b) and DU145 cells (not shown). Interestingly, while bicalutamide alone at lower doses (e.g., 10–20 μM) had no effect on androgen-independent prostate cancer cell growth inhibition, combination of curcumin with bicalutamide showed a significant inhibition of cell growth in not only androgen-dependent but also androgen-independent prostate cancer cells (Fig. 1c). This suggested a potential new mechanism by which the combination of curcumin and bicalutamide enhanced the growth inhibition in androgen-independent prostate cancer cells.

### Curcumin and bicalutamide increased phosphorylation of ERK1/2 and SAPK/JNK

**Fig. 2:**
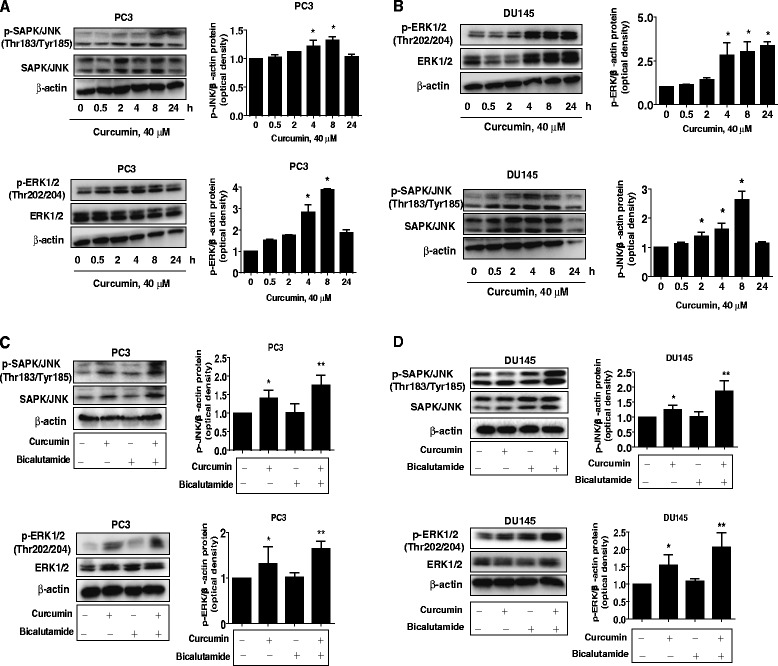
Curcumin and bicalutamide increased phosphorylation of ERK1/2 and SAPK/JNK. **a-b** PC3 and DU145 cells were treated with curcumin (40 μM) in the indicated times, and cell lysate was harvested and the expression of the phosphorylated or total protein of ERK1/2 and SAPK/JNK were measured by Western blot analysis using corresponding antibodies. GAPDH was used as loading control. **c-d** PC3 and DU145 cells were treated with curcumin (40 μM) and bicalutamide (30 μM) for 8 h. Afterwards, the phosphorylation and expression of ERK1/2 and SAPK/JNK were detected by Western blot. Values in bar graphs were given as the mean ± SD from three independent experiments performed in triplicate. *indicates significant difference as compared to the untreated control group (P < 0.05). **Indicates significant difference from curcumin treated alone (P < 0.05)

We then explored the potential signaling that may be involved in the inhibitory response by curcumin and bicalutamide in androgen-independent prostate cancer cells. We showed that curcumin increased the phosphorylation of ERK1/2 and SAPK/JNK in a time-dependent fashion with significant induction observed at 4–8 h in PC3 and DU145 cells (Fig. 2a, b). Furthermore, combination of curcumin with bicalutamide further enhanced the phosphorylation of ERK1/2 and SAPK/JNK in PC3 and DU145 cells (Fig. 2c, d).

### The effect of curcumin and bicalutamide on protein expression of p65 and MUC1-C through activation of MEK/ERK/12 and SAPK/JNK

**Fig. 3:**
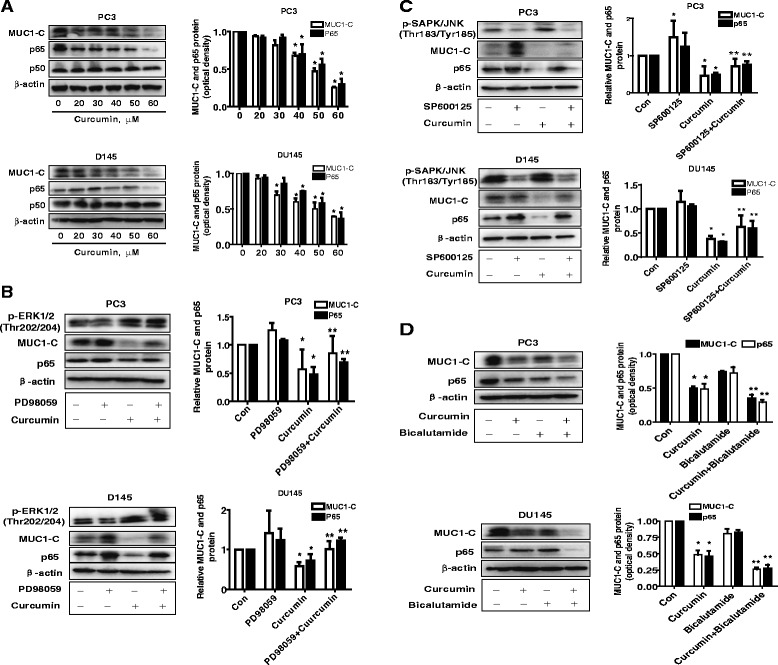
The effect of curcumin and bicalutamide on protein expression of p65 and MUC1-C through activation of ERK/12 and SAPK/JNK. **a** PC3 and DU145 cells ells were exposed to increased concentration of curcumin for 24 h. Afterwards, the expression of p65 and MUC1-C proteins was detected by Western blot. **b-c** PC3 and DU145 cells were treated with PD98059 (10 μM) and SP600125 (20 μM) for 2 h before exposure of the cells to curcumin (40 μM) for an additional 24 h. Afterwards, the expression of p65 and MUC1-C protein were detected by Western blot using antibodies against p65 and MUC1-C. The bar graphs represent the mean ± SD of p65 or MUC1-C /GAPDH of three independent experiments. **d,** PC3 and DU145 cells were treated with curcumin (40 μM) and bicalutamide (30 μM) for 24 h. Afterwards, the expression of p65 and MUC1-C protein were detected by Western blot using antibodies against p65 and MUC1-C. Values in bar graphs were given as the mean ± SD from three independent experiments performed in triplicate. *indicates significant difference as compared to the untreated control group (P < 0.05). **Indicates significant difference from curcumin treated alone (P < 0.05)

Next, we further examined the potential molecular mechanism affected by curcumin in the presence or absence of bicalutamide. We showed that curcumin reduced the protein expression of p65, NF-κB subunit, and MUC1-C, a membrane-anchored mucin, in the dose-dependent manner in PC3 and DU145 cells (Fig. 3a). Note that curcumin had no effect on p50 protein expression (Fig. 3a). Interestingly, the inhibitors of MEK/ERK1/2 (PD98059) and SAPK/JNK (SP60015) blocked the effect of curcumin on both p65 and MUC1-C protein expression (Fig. 3b, c). More importantly, we found that combination of curcumin with bicalutamide further significantly reduced the protein expression of p65 and MUC1-C suggesting a synergy in this process (Fig. 3d).

### Exogenous expression of p65 abrogated the effect of curcumin on MUC1-C expression and cell growth inhibition

**Fig. 4:**
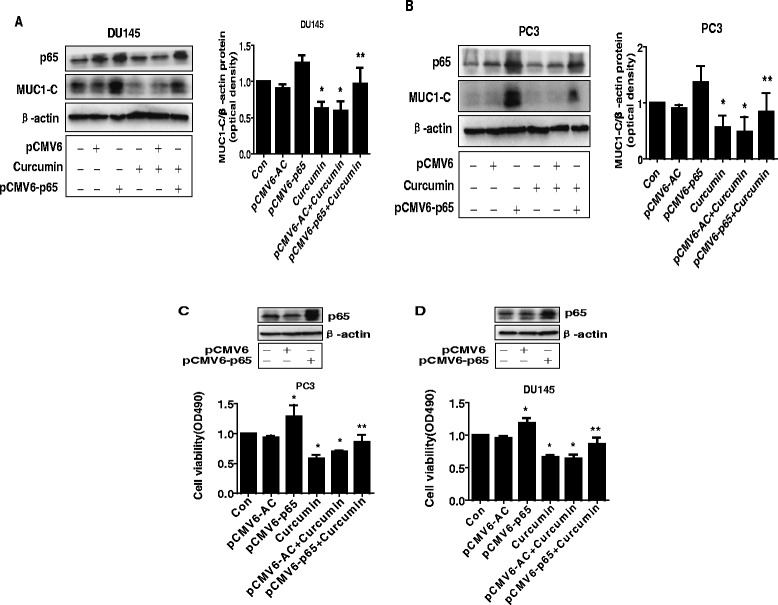
Exogenous expression of p65 abrogated the effect of curcumin on MUC1-C expression and cell growth inhibition. **a-b** PC3 and DU145 cells were transfected with the control (pCMV6) or expression constructs of p65 for 24 h before exposing the cells to curcumin for an additional 24 h. Afterwards, p65 and MUC1-C protein expression were determined by Western blot. **c-d** PC3 and DU145 cells were transfected with the control (pCMV6) or expression construct of p65 for 24 h before exposing the cells to curcumin for an additional 48 h. Afterwards, the cell viability was determined using the MTT assay as described in the Materials and Methods section and was expressed as percentage of control in the mean ± SD of three separate experiments. Insert on the upper panel represented the protein levels of p65 as determined using Western blot. β-actin was used as internal control. Values in bar graphs were given as the mean ± SD from three independent experiments performed in triplicate. *Indicates significant difference as compared to the untreated control group (P < 0.05). **Indicates significant difference from curcumin treated alone (P < 0.01)

Moreover, we tested the role of p65 in this process; we showed that exogenous expression of p65 blocked the effect of curcumin on MUC1-C expression in PC3 and DU145 cells (Fig. 4a, b); this suggested that p65 is an upstream of MUC1-C. In addition, we found that overexpression of p65 reversed curcumin-inhibited growth in PC3 and DU145 cells (Fig. 4c, d).

### While overexpression of MUC1-C had no effect on p65, it attenuated the effect of curcumin on cell growth inhibition and phosphorylation of ERK1/2 and SAPK/JNK

**Fig. 5:**
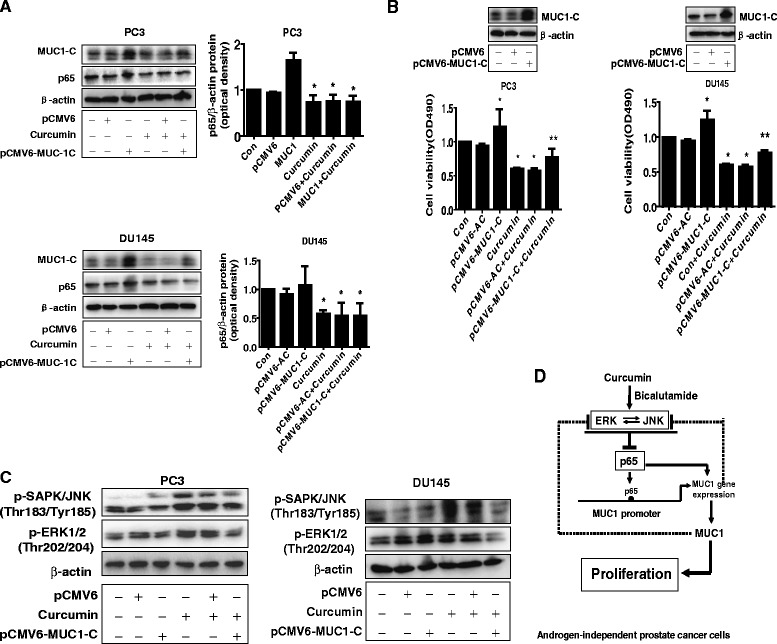
Overexpression of MUC1-C attenuated the effect of curcumin on cell growth inhibition and curcumin-induced phosphorylation of ERK1/2 and SAPK/JNK. **a** PC3 and DU145 cells were transfected with the control (pCMV6) or expression constructs of MUC1-C for 24 h before exposing the cells to curcumin for an additional 24 h. Afterwards, p65 and MUC1-C protein expression were determined by Western blot. **b** PC3 and DU145 cells were transfected with the control (pCMV6) or expression constructs of MUC1-C for 24 h before exposing the cells to curcumin for an additional 48 h. Afterwards The cell viability was determined using the MTT assay as described in the Materials and Methods section. Insert on the upper panel represented the protein levels of MUC1-C as determined by Western blot. β-actin was used as internal control. Values in bar graphs were given as the mean ± SD from three independent experiments performed in triplicate. *Indicates significant difference as compared to the untreated control group (P < 0.05). **Indicates significant difference from curcumin treated alone (P < 0.01). **c** PC3 and DU145 cells were transfected with the control (pCMV6) or expression constructs of MUC1-C for 24 h before exposing the cells to curcumin for an additional 8 h. Afterwards, p-SAPK/JNK and p-ERK1/2 were determined by Western blot. **d** The diagram the shows that curcumin inhibits the growth of androgen-independent prostate cancer cells through ERK1/2 and SAPK/JNK-mediated inhibition of p65, followed by reducing expression of MUC1-C. There is a synergy of curcumin and bicalutamide. The negative feedback regulatory loop of MUC1-C to ERK1/2 and SAPK/JNK signaling pathways also adds the important role of MUC1-C in mediating the overall responses of curcumin

To further determine the role of MUC1-C in this process, we transfected the exogenous expression vector of MUC1-C into the cells and found that, while overexpression of MUC1-C had no effect on curcumin-reduced p65 protein expression in PC3 and DU145 cells (Fig. 5a), it significantly antagonized the effect of curcumin on cell growth inhibition (Fig. 5b). This result indicated the critical role of MUC1-C in this process. Interestingly, exogenous expression of MUC1-C significantly attenuated the curcumin-induced phosphorylation of SAPK/JNK and ERK1/2 in PC3 and DU145 cells (Fig. 5c).

## Discussion

Majority of advanced prostate cancers are sensitive to androgen deprivation therapy in the first beginning, then subsequently progress to the CRPC. The reasons remain unknown. Because of the limitations of current therapeutic approaches, many patients die of recurrent and metastatic diseases. The combination with anti-inflammatory and other adjuvant therapies present a very promising treatment approach for this malignancy. Curcumin is a promising anticancer agent for various cancer types including prostate cancer cells, and involves in multiple signaling and potential targets [6, 7, 19]. However, the detailed molecular mechanisms underlining suppression of androgen-independent prostate cancer cell growth still remain to be elucidated. In this study, we demonstrated not only a significant inhibition of growth of prostate cancer cells by curcumin, but more importantly, a synergistic effect observed in combination of curcumin with bicalutamide, an androgen receptor antagonist, in androgen-independent prostate cancer cells. These findings implied a potential new mechanism for this synergy that was AR-unrelated. The molecular mechanisms by which curcumin inhibited growth of androgen-dependent and -independent prostate cancer cells were reported in other studies demonstrating that pathways and transcription factors other than AR-mediated and -regulated downstream genes were involved in this process [20–22]. However, more experiments are needed to further determine this.

In this study, we demonstrated the role of SAPK/JNK and MEK/ERK1/2 signaling pathways in mediating the effect of curcumin and bicalutamide on controlling growth of androgen-independent prostate cancer cells. The activation of these kinases by curcumin or/and bicalutamide has been shown in other studies implicating in the regulation of other gene expression and subsequent cellular responses [23–28]. Consistent with ours, one report showed that activation of SAPK/JNK was involved in the curcumin-triggered intrinsic apoptotic pathway in cardiac myoblasts [23]. Also, activation of MEK/ERK1/2 pathway mediated in curcumin-induced cell cycle arrest and apoptosis in human gastric cancer cells [25]. We noticed that opposite results were also reported in other cell system [29]. It was possible that the different environment contexts, cell lines used, and other unknown factors were account for this discrepancy. Thus, more studies are required to further confirm this. Findings from other studies including gene deletion data indicated the conflicting results in essence that turn MEK/ERK1/2 and SAPK/JNK functions from oncogene to tumor suppressor, which could suggest the possible dual functions of these kinases (pro-oncogene or tumor suppressor) [30–32]. Therefore, the insight true role of SAPK/JNK and MEK/ERK1/2 signaling pathways in triggering cancer cell differentiation, senescence, apoptosis, and survival appeared to be context dependent and more complicated, which needs to be clarified in the future.

To further explore the potential mechanism underlining the aforementioned, we tested the involvement of MUC1-C. Our results indicated the critical role of MUC1-C in mediating the effect of curcumin on inhibition of growth of androgen-independent prostate cancer cells. Consistent with this, other studies were observed the similar findings and suggested that MUC1 including its subunits (e.g., MUC1-C) could be a potential target of curcumin in the treatment of prostate and breast cancer cells [33, 34]. Furthermore, we demonstrated an important role of NF-κB/p65 that may involve in the effects of curcumin on MUC1-C expression and prostate cancer cell growth. Curcumin was shown to be a strong inhibitor of NF-κB activity and its inhibitory effect on NF-κB related pathways led to enhance the cytotoxicity of chemotherapeutic agents in prostate cancer cells [35]. Moreover, reported data have demonstrated the link of MUC1-C and NF-κB signaling in other studies [36, 37]. Cancer cells are dependent on intact MUC1-C function for constitutive activation of the canonical NF-κB pathway and cancer cell growth, and survival [36, 37]. MUC1-C is a direct activator of NF-κB/p65 and that an inhibitor of MUC1 function is effective in blocking activation of the NF-κB pathway in nonmalignant epithelial cells [36, 37]. Our results implied that MUC1-C was downstream of NF-κB. Consistent with this, early study showed that MUC1 promoter region contained NF-κB/p65 binding site that mediated the proinflammatory cytokines-induced MUC1 promoter activity and gene expression in normal breast epithelia and breast cancer cells [38]. Thus, the function and regulation of NF-κB/p65-MUC1-C complexes are more complicated than we could think. Nevertheless, we reasoned that regulation of p65 played a crucial role in mediating curcumin-inhibited MUC1-C expression in androgen-independent prostate cancer cells.

We also observed the involvement of activation of MEK/ERK1/2 and SAPK/JNK signaling pathways in the regulation of p65 and MUC1-C affected by curcumin. The links of these kinases to the regulation of NF-κB and MUC1-C have been shown in other studies, suggesting that blockade of these kinases could reduce expression of MUC1 in several cell systems [12, 39–41]. More interestingly, we also showed the negative feedback regulation of ERK1/2 and SAPK/JNK by MUC1-C. These signaling axes formed a bidirectional feedback loop to mediate curcumin-inhibited prostate cancer proliferation. The feedback regulation circuit of kinases with other genes/proteins was reported in other studies demonstrating relative common autocrine or paracrine physiopathological phenomena [42, 43]. One study found that sustained activation of SAPK/JNK inhibited NF-κB signaling via a feedback loop mechanism that led to an alteration in the transcription of the NF-κB-induced apoptotic gene in immortalized renal proximal tubular epithelial cells [44]. More experiments are warranted to better elucidate the in-depth mechanism for these unexplored complicated regulatory circuit.

More importantly, we demonstrated a synergy of combination of curcumin and bicalutamide, the androgen receptor antagonist, in the inhibition of p65, MUC1-C and androgen-independent prostate cancer cell growth, implying that signaling and mechanisms other than through the AR-mediated regulatory pathways and genes contributed to the overall enhanced effects. On the other hand, this also suggested the potential new mechanism of bicalutamide in controlling androgen-independent prostate cancer cell growth. Bicalutamide was found to inhibit androgen-independent prostate cancer cell growth appeared through AR-independent pathways [45, 46]. One study showed that combination of bicalutamide with other therapeutic agents enhanced the CRPC growth inhibition via up-regulation of insulin-like growth factor-binding protein 3 (IGFBP3) [47]. Also, previous study indicated that MUC1-C subunit suppressed AR expression through a posttranscriptional mechanism and resisted to bicalutamide treatment in androgen-dependent prostate cancer cells, this implied that inhibition of AR expression by MUC1-C led to develop more aggressive androgen-independent phenotype in prostate cancer cells that was sensitive to MUC1-C inhibition [10]. Consequently, this may facilitate the therapeutic effects of bicalutamide. We reasoned that MUC1-C could be a potential target of curcumin in suppression of androgen-independent prostate cancer cell growth, and inhibition of MUC1-C by curcumin sensitized the therapeutic effect of bicalutamide in prostate cancer cell growth. A cell based morphology experiment showed that curcumin analogs or curcumin-anti-androgen conjugates demonstrated more potent than bicalutamide alone in the cytotoxic effects on LNCaP and PC-3 cells through suppression of pseudopodia formation, which was highly related to cell migration and tumor metastasis, other than targeting AR [48]. Also, note that studies implicated the dissected mechanisms by which using different anti-androgen receptor compounds on affecting prostate cancer invasion and metastasis, resulting in opposite effects [49]; the potential risks of using these agents, such as bicalutamide, among others, on prostate cancer metastasis require to be carefully evaluated. Thus, additional studies are warranted to further explore the combination (curcumin and bicalutamide) effects on invasion and metastasis of androgen-independent prostate cancer cells.

## Conclusion

This study shows that curcumin inhibits the growth of androgen-independent prostate cancer cells through MEK/ERK1/2 and SAPK/JNK-mediated inhibition of p65, followed by reducing expression of MUC1-C protein. More importantly, there is a synergy of curcumin and bicalutamide. The negative feedback regulatory loop of MUC1-C to ERK1/2 and SAPK/JNK signaling pathways further added the important role of MUC1-C in mediating to the overall responses of curcumin (Fig. 5d). This study unveils the potential molecular mechanism by which combination of curcumin and bicalutamide enhances the growth inhibition of androgen-independent prostate cancer cells.

## Abbreviations

MUC1: Cell surface-associated mucin 1
MUC1-C: MUC1-C-terminal subunit
ADT: Androgen deprivation therapy
AR: Androgen receptor
CRPC: Castration resistant prostate cancer
ERK1/2: Extracellular regulated protein kinase 1/2
MEK: MAPK extracellular signaling-regulated kinase (ERK) kinase
SAPK/JNK: Stress-activated protein kinase/c-Jun N-terminal kinase
NF-κB: Nuclear factor kappa β
IGFBP3: Insulin-like growth factor-binding protein 3
